# Polymeric Biomaterials for the Delivery of Stem Cell-Derived Exosomes in Inflammatory Skin Diseases: Engineering Strategies and Synergistic Effects

**DOI:** 10.3390/polym18141781

**Published:** 2026-07-21

**Authors:** Myungkyung Noh, Tae-Hyun Heo, Min-Kyu Kang, Gun-Jae Jeong

**Affiliations:** 1 Institute of Molecular Biology and Genetics, Seoul National University, Seoul 08826, Republic of Korea; 2 School of Nanomedical Engineering, Korea National University of Transportation, Chungju-si 27469, Republic of Koreaalsrb6899@ut.ac.kr (M.-K.K.)

**Keywords:** stem cell-derived exosomes, polymeric biomaterials, inflammatory skin diseases, skin tissue engineering, smart polymeric systems

## Abstract

Skin tissue engineering has emerged as a promising therapeutic strategy for severe wounds and inflammatory skin diseases. Stem cell-derived exosomes (SC-Exos) have recently gained increasing attention as cell-free therapeutic agents with regenerative and immunomodulatory potential, offering possible advantages over direct stem cell transplantation. To fully realize their therapeutic potential, however, efficient delivery platforms are needed to enhance local retention, preserve vesicle integrity, and support sustained release within the diseased skin microenvironment. In this review, we discuss advanced polymeric biomaterials as functional delivery platforms for SC-Exos in skin tissue engineering. We focus on natural and synthetic polymers engineered into nanofibrous scaffolds, hydrogels, and microneedles, and examine how these systems enhance exosome loading, protect vesicle integrity, improve local retention, and modulate release kinetics. We further highlight the therapeutic effects and underlying mechanisms of polymer–exosome systems in skin lesion repair, focusing on their roles in promoting angiogenesis, modulating local inflammation and immune responses, and facilitating extracellular matrix remodeling. Finally, we address remaining challenges and future directions for translating polymer-based SC-Exos delivery platforms into clinically relevant skin regenerative therapies.

## 1. Introduction

Inflammatory skin diseases (ISDs), including atopic dermatitis (AD) and psoriasis, are a group of disorders characterized by chronic, aberrant inflammatory responses in the skin [1]. Furthermore, severe cutaneous injuries, such as chronic or diabetic wounds, frequently stall in a prolonged inflammatory phase. Because these non-healing wounds share fundamental pathological hallmarks with classic ISDs, including unresolved inflammation, elevated oxidative stress, and severely impaired barrier function, they are equally critical targets for advanced immunomodulatory and regenerative nanotherapeutics. Patients with ISDs often experience chronic pain, pruritus, social stigma, and impaired quality of life, which collectively contribute to a substantial global health burden [2,3]. Conventional treatments for ISDs, including topical corticosteroids, calcineurin inhibitors, and systemic immunosuppressants, are widely used to control excessive inflammation (Table 1) [4,5]. However, these therapies primarily provide symptomatic control, and their long-term or extensive use can cause local and systemic adverse effects, including skin atrophy, glaucoma, hyperglycemia, and hypertension [4,5,6]. Recently, stem cell-derived exosomes (SC-Exos) have emerged as a paradigm-shifting nanotherapeutic [7]. As lipid-bound extracellular vesicles (30–150 nm in diameter), SC-Exos carry a rich cargo of regulatory microRNAs, proteins, and lipids that not only orchestrate immunomodulation but also accelerate tissue regeneration without the tumorigenic or immunogenic risks associated with direct stem cell transplantation [7,8,9]. Unlike conventional therapies for ISDs that primarily dampen inflammatory signaling, SC-Exos can modulate the local immune microenvironment while promoting keratinocyte migration, fibroblast activation, angiogenesis, and extracellular matrix remodeling, thereby actively supporting restoration of skin homeostasis [10,11,12]. Despite their enormous biological potential, the clinical translation of SC-Exos is severely hindered, because of their poor retention at the application site and susceptibility to enzymatic degradation, especially within the harsh inflammatory microenvironments of diseased skin [13,14,15]. To overcome these physiological barriers, advanced polymeric biomaterials have emerged as promising delivery vehicles for SC-Exos, offering enhanced target-site retention, controlled release, and preservation of exosomal structural integrity and biological activity [7]. To this end, this review provides a comprehensive analysis of polymeric systems engineered for SC-Exos encapsulation and delivery. By focusing on the structural design, physicochemical properties, and stimuli-responsive features of natural and synthetic polymers, we highlight how polymer engineering can protect SC-Exos within inflamed tissues, improve their pharmacokinetic properties, and ultimately enhance their capacity to resolve inflammation and promote skin regeneration. Unlike generic reviews on exosome delivery or basic hydrogel fabrication, this review explicitly delineates the critical distinction between mere additive effects and true synergistic mechanisms in polymer–exosome platforms. Furthermore, we focus heavily on the macromolecular chemistry of ‘smart’ (stimuli-responsive) polymers to provide a forward-looking perspective on next-generation dermatological treatments. During the preparation of this review manuscript, generative AI technologies were utilized to assist in structuring the text, improving language fluency, and formatting the manuscript. Detailed information regarding the specific AI tools used is provided in the Acknowledgments Section.

## 2. Pathophysiological Microenvironment of Inflammatory Skin

**Figure 1:**
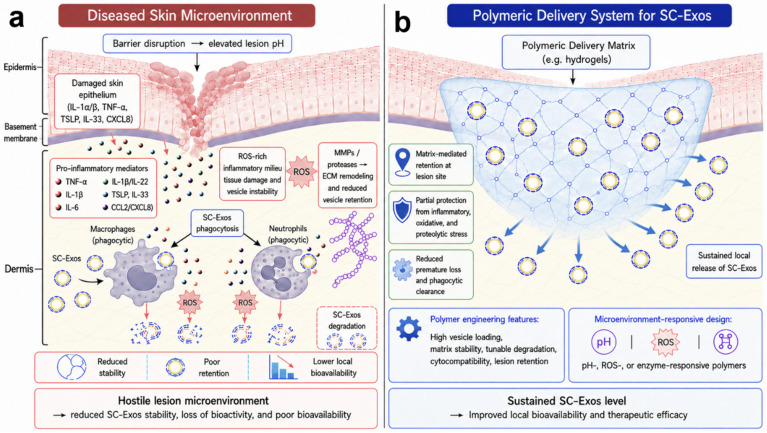
**Physical and biochemical landscape of diseased skin and polymeric delivery strategies for effective SC-Exos delivery.** (**a**) The skin microenvironment of ISDs is often characterized by barrier disruption and chronic inflammation. Disruption of skin barrier increases lesion pH and inhibits protective mechanisms against external and endogenous insults. Pro-inflammatory cytokines and chemokines are released from damaged skin epithelium, promoting the migration of immune cells, including phagocytic macrophages and neutrophils, to the lesion site. These recruited immune cells further amplify inflammatory responses by releasing pro-inflammatory mediators, ROS, and ECM-remodeling factors such as MMPs and proteases, which collectively lead to persistent inflammation. This hostile microenvironment can promote biochemical degradation and phagocytic clearance of SC-Exos, reduce their local bioavailability, and ultimately compromise therapeutic performance of SC-Exos in inflamed skin. (**b**) Polymeric delivery matrices represent a potential engineering strategy for effective SC-Exos delivery to inflammatory skin lesions. Topical delivery of SC-Exos encapsulated within polymeric matrices can reduce burst release and phagocytic clearance, thereby enhancing vesicle retention at the lesion site and protecting SC-Exos from oxidative and proteolytic stress. Beyond conventional matrix engineering, smart polymers that respond to local microenvironmental cues, including elevated pH, ROS, and proteolytic enzymes, can further sustain local SC-Exos levels. Collectively, these polymeric delivery systems improve the local bioavailability and therapeutic efficacy of SC-Exos in inflamed skin.

Understanding the physical and biochemical landscape of diseased skin is essential for designing effective polymeric delivery systems that can enhance the therapeutic performance of SC-Exos. Despite their diverse etiologies, ISDs commonly develop within a pathological microenvironment characterized by chronic inflammation, epidermal barrier disruption and associated pH dysregulation, oxidative stress by excessive reactive oxygen species (ROS), protease-mediated extracellular matrix (ECM) remodeling [39,40,41]. Notably, this hostile microenvironment is not exclusive to autoimmune or allergic dermatoses. Chronic wounds similarly represent a state of stalled healing driven by an unrelenting inflammatory phase and a highly proteolytic, ROS-enriched milieu. Consequently, polymeric delivery platforms engineered to modulate the ISD microenvironment are highly translatable and therapeutically relevant for chronic wound management. These disease-associated features can substantially limit the therapeutic efficacy of SC-Exos by accelerating their degradation, clearance, and loss of bioactivity (Figure 1) [42,43].

Chronic inflammation is a central feature of the ISD microenvironment. Although the dominant immune pathways differ across ISDs, including type 2 inflammation in AD and IL-23/IL-17 axis activation in psoriasis, these conditions commonly share a cytokine-rich inflammatory milieu [44,45]. In ISD lesion, infiltrating immune cells produce elevated levels of pro-inflammatory cytokines (e.g., TNF-α, IL-1β, IL-6, IL-17, and IL-22), while activated keratinocytes and fibroblasts release cytokines, chemokines, and matrix-remodeling mediators (e.g., TSLP, IL-33, CCL2, CCL20, CXCL8, and matrix metalloproteinases [MMPs]) that further recruit leukocytes and amplify local inflammation [46,47,48,49,50]. Collectively, these processes disrupt epidermal homeostasis, weaken barrier integrity, and promote persistent tissue damage.

Barrier disruption in inflamed skin is closely associated with local biochemical alterations, including pH dysregulation. Healthy skin maintains a slightly acidic surface environment (pH 4.5–5.5), which supports barrier integrity, antimicrobial defense, and enzyme homeostasis [51]. In chronic inflammatory lesions, particularly in AD, impaired barrier function and microbial colonization often shift the skin surface toward a more alkaline state [52,53]. This altered pH environment may affect SC-Exos stability, polymer degradation kinetics, and cargo release behavior, making pH an important design parameter for polymeric delivery systems. In parallel, activated immune cells generate excessive ROS, creating an oxidative microenvironment that damages proteins and lipids of both ECM and cells, which can also destabilize lipid bilayers and membrane-associated proteins of SC-Exos [27,53]. Inflammatory skin lesions also exhibit increased proteolytic activity, including overexpression of MMPs, which contributes to ECM degradation, tissue remodeling, and premature breakdown of biologic therapeutics or delivery matrices [54,55]. Moreover, abundant phagocytic cells within inflamed skin, such as macrophages, dendritic cells, and neutrophils, can rapidly internalize extracellular vesicles, thereby reducing the availability of SC-Exos to target skin-resident cells [56]. Together, oxidative stress, proteolytic degradation, phagocytic uptake, and ECM instability can expose freely administered SC-Exos to rapid damage and clearance, resulting in poor local retention and a shortened therapeutic window.

In this context, polymeric delivery systems can function as protective reservoirs that physically retain SC-Exos within the lesion, shield them from immediate exposure to the hostile inflammatory microenvironment, and regulate their gradual release through diffusion- or degradation-controlled mechanisms [57,58,59]. Such sustained release may help maintain therapeutically relevant SC-Exos levels over time while reducing burst loss, premature degradation, and rapid phagocytic clearance [60,61,62]. To achieve these outcomes, polymer matrices that can efficiently encapsulate SC-Exos are required while providing mechanical stability, tunable degradation kinetics, and biocompatibility within inflamed tissues. Therefore, the therapeutic performance of SC-Exos delivery systems is closely linked to polymer engineering strategies, including backbone design to modulate physicochemical properties and the incorporation of microenvironment-responsive features that respond to pathological cues in inflamed skin.

## 3. Polymeric Biomaterials for SC-Exos Encapsulation and Delivery

**Figure 2:**
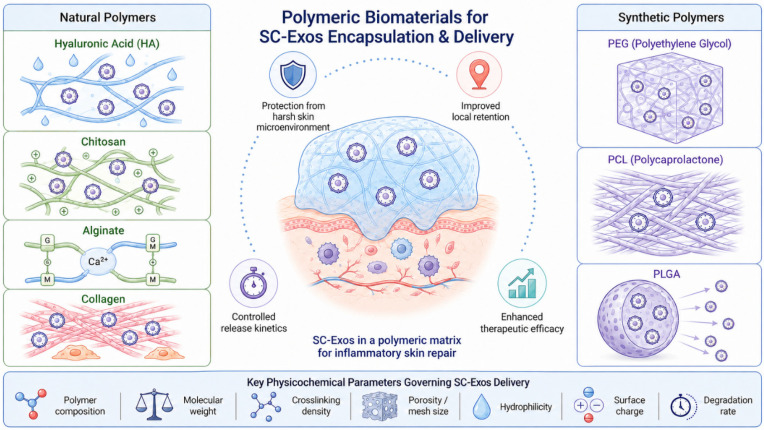
**Types of polymeric biomaterials and key engineering parameters for optimal SC-Exos encapsulation and delivery for inflammatory skin repair.** Polymeric biomaterials for SC-Exos delivery can be broadly classified into natural and synthetic polymers. Natural polymers, including HA, chitosan, alginate, and collagen, are widely used for skin tissue engineering because of their biocompatibility and physicochemical resemblance to the skin ECM. Synthetic polymers, such as PEG, PCL, and PLGA, offer greater tunability, reproducibility, and engineering flexibility enabling precise control over mechanical properties, vesicle retention, and release kinetics. Key physicochemical parameters should be considered to improve local bioavailability and regulate release kinetics, thereby optimizing the therapeutic efficacy of SC-Exos.

The sustained release behavior of SC-Exos is fundamentally governed by the physicochemical interaction forces between the polymer matrix and the vesicles. Beyond simple steric entrapment within the polymer mesh, electrostatic interactions play a pivotal role. For example, the negatively charged lipid bilayer of exosomes forms strong ionic bonds with cationic polymers like chitosan. Additionally, modifying polymers with specific affinity ligands enables non-covalent, reversible tethering. These interaction forces prevent the initial burst release and allow degradation-mediated, zero-order release kinetics. Polymeric biomaterial-mediated encapsulation can protect SC-Exos from the harsh microenvironment of diseased skin while enhancing their delivery efficiency and therapeutic performance through improved local retention and controlled release kinetics [61,62]. These outcomes are strongly governed by the choice of base polymer, which dictates the structural integrity, release kinetics, and biological performance of the exosome-polymer composite. Key physicochemical parameters, including polymer composition, molecular weight, crosslinking density, porosity, hydrophilicity, and surface charge, can influence SC-Exos loading, stability, diffusion, and release [63]. Thus, selecting an appropriate polymer backbone is essential for optimizing SC-Exos delivery. Polymers used for SC-Exos encapsulation are generally categorized as natural or synthetic polymers, each with distinct properties that shape their suitability for inflammatory skin applications (Figure 2) [59]. While lipid-based nanocarriers, such as liposomes and lipid nanoparticles (LNPs), are currently at the forefront of intracellular drug delivery, they inherently lack the mechanical integrity required to serve as macroscopic tissue dressings. In the context of inflammatory skin diseases (ISDs) and severe wounds, the delivery vehicle must do more than simply transport the nanovesicles; it must provide robust structural support, manage wound exudates, and offer physical barrier protection against secondary infections. Polymeric biomaterials uniquely bridge this gap. Unlike delicate lipid vesicles, polymers can be engineered into versatile macroscopic formats, such as hydrogels, electrospun scaffolds, and microneedles, with highly tunable rheological and mechanical properties [64,65,66,67]. This macroscopic adaptability makes polymeric systems exceptionally suited to cover extensive epidermal lesions, retain essential tissue moisture, and conform seamlessly to irregular wound beds, thereby creating an optimal physical and biochemical microenvironment for SC-Exos-mediated regeneration.

### 3.1. Natural Polymers

Natural polymers are biomacromolecules derived from biological sources, such as polysaccharides and proteins, and are widely used as delivery matrices for skin tissue engineering because of their inherent biocompatibility, biodegradability, and structural resemblance to the native ECM [68,69]. The skin ECM is a hydrated, dynamic network composed of fibrous proteins, glycosaminoglycans, proteoglycans, and adhesive molecules that provide mechanical support, regulate cell adhesion and migration, and modulate local biochemical signaling during inflammation and repair [70,71,72]. Owing to these ECM-like features, natural polymers can provide a favorable microenvironment for SC-Exos encapsulation and delivery by supporting vesicle stability, maintaining tissue compatibility, and minimizing adverse immune responses [63]. In inflammatory skin applications, natural polymers are particularly attractive because they can form hydrogels or scaffolds that conform to irregular lesions, retain moisture, allow diffusion of bioactive vesicles, and support tissue repair [73]. Representative natural polymers discussed here include hyaluronic acid (HA), chitosan, alginate, and collagen, each of which offers distinct physicochemical and biological properties for SC-Exos delivery.

#### 3.1.1. Hyaluronic Acid (HA)

As a ubiquitous glycosaminoglycan in the skin, HA regulates hydration, tissue repair, and extracellular homeostasis [74]. Owing to its abundant hydroxyl and carboxyl groups, HA is highly hydrophilic, making HA-based hydrogels well suited for inflammatory skin lesions characterized by impaired barrier function and increased transepidermal water loss [71]. For SC-Exos delivery, HA-based hydrogels can physically entrap vesicles within a hydrated polymer network, thereby reducing burst degradation and premature clearance [75,76,77]. By tuning HA concentration, molecular weight, crosslinking density, and mesh size, these hydrogels can further regulate vesicle diffusion, local retention, and sustained release [78]. Thus, HA-based hydrogels may function as hydration-supporting matrices that promote skin barrier restoration and protective reservoirs that improve SC-Exos availability within inflamed skin [79,80,81,82,83].

#### 3.1.2. Chitosan

Chitosan, a cationic polysaccharide derived from deacetylated chitin, is particularly attractive for SC-Exos delivery because its positive charge enables electrostatic interactions with negatively charged exosomal membranes [84]. These interactions can enhance vesicle loading and retention within the polymer matrix, restrict diffusion-driven burst release, and promote sustained SC-Exos release. Beyond its role as a delivery scaffold, chitosan also provides intrinsic antibacterial support through interactions between its protonated amino groups and negatively charged bacterial membranes, which can disrupt membrane integrity and suppress bacterial colonization [85,86,87]. Thus, chitosan-based hydrogels are well suited for barrier-disrupted inflammatory skin lesions, where they can simultaneously improve SC-Exos retention and release kinetics while reducing microbial burden that can exacerbate local inflammation [88,89,90,91,92].

#### 3.1.3. Alginate

Alginate, an anionic polysaccharide derived from brown algae, is composed of β-D-mannuronic acid (M) and α-L-guluronic acid (G) residues. Its key advantage for SC-Exos delivery is its ability to rapidly form hydrogels through Ca^2+^-mediated ionic crosslinking of G-rich blocks, allowing vesicles to be physically entrapped within the polymer network while avoiding extensive backbone modification or reactive crosslinking chemistry that may compromise vesicle integrity [93,94]. Because G-rich alginates form stiffer and denser networks, whereas M-rich regions confer greater flexibility, the G/M ratio provides a useful handle for tuning alginate-based hydrogel mechanics and architecture [61,95]. By adjusting alginate concentration, molecular weight, Ca^2+^ concentration, and the G/M ratio, alginate hydrogels can provide tunable control over matrix stiffness and porosity, thereby regulating SC-Exos diffusion, local retention, and burst release at damaged skin sites [95]. Thus, alginate is particularly valuable for SC-Exos delivery regarding its simple ion-triggered encapsulation and controllable retention–release behavior [90,94,96,97,98,99,100,101,102,103].

#### 3.1.4. Collagen

Collagen, the major structural protein of the skin ECM, offers distinct advantages for SC-Exos delivery by providing a native fibrillar architecture and a cell-adhesive microenvironment [104]. Through intrinsic integrin-binding motifs, collagen supports interactions with keratinocytes, fibroblasts, and endothelial cells, thereby promoting cell adhesion, migration, re-epithelialization, fibroblast activity, angiogenesis, and ECM remodeling [91,105]. Similar to other natural polymer-based matrices, collagen-based hydrogels or scaffolds can physically entrap vesicles within a fibrillar ECM-like network, while tunable collagen concentration, fibril density, and crosslinking degree can regulate SC-Exos diffusion, local retention, and sustained release [91,106]. Thus, collagen-based matrices may function not only as protective reservoirs that prolong SC-Exos availability within inflamed skin but also as bioactive ECM scaffolds that actively guide cellular processes required for skin regeneration [107,108].

### 3.2. Synthetic Polymers

While natural polymers provide intrinsic biocompatibility, tissue-like architecture, and bioactive cues, synthetic polymers complement natural polymers by offering greater tunability, reproducibility, and engineering flexibility. Their chemical composition, molecular weight, crosslinking density, degradation rate, and functional groups can be precisely controlled, enabling predictable regulation of mechanical properties and SC-Exos release kinetics [60]. In addition, synthetic polymers can be engineered as stimuli-responsive or biofunctionalized matrices, allowing exosome retention and release to be tailored to the microenvironment of diseased skin [109]. Synthetic polymeric materials used in skin engineering include poly(ethylene glycol) (PEG), poly(ε-caprolactone) (PCL), poly(lactic-co-glycolic acid) (PLGA), poly(vinyl alcohol) (PVA), polyurethane (PU), and poly(lactic acid)-based polymers [110,111]. In this section, we focus on PEG, PCL, and PLGA, as these polymers represent three major design strategies for SC-Exos delivery: hydrogel-based retention, scaffold-based structural support, and degradation-controlled sustained release. Furthermore, polymers such as PVA and poly(*N*-isopropylacrylamide) (PNIPAM) are highly valued for their exceptional structural stability and temperature-responsive sol–gel transition capabilities, respectively.

#### 3.2.1. Poly(ethylene glycol) (PEG)

PEG is one of the most widely used synthetic polymers for hydrogel-based drug delivery owing to its hydrophilicity, biocompatibility, and low nonspecific protein adsorption. As a bioinert polymer, PEG provides a well-defined and highly tunable matrix that can physically encapsulate SC-Exos without introducing undefined biological cues. The molecular weight, polymer concentration, and crosslinking density of PEG hydrogels can be adjusted to regulate mesh size, swelling behavior, mechanical stiffness, and vesicle diffusion, thereby enabling controlled local retention and sustained release of SC-Exos [112,113]. In addition, PEG can be chemically functionalized with cell-adhesive peptides, degradable linkers, or ECM-mimetic motifs to overcome its limited intrinsic bioactivity [114]. Therefore, PEG-based hydrogels are particularly useful when reproducible, minimally bioactive, and precisely tunable delivery platforms are required [112,113,115].

#### 3.2.2. Poly(ε-caprolactone) (PCL)

PCL is a biodegradable aliphatic polyester widely used for structural scaffold fabrication owing to its mechanical stability, processability, and slow degradation. Its hydrophobicity contributes to limited water uptake and delayed hydrolytic degradation, thereby helping PCL scaffolds maintain their structural integrity over extended periods. In the context of SC-Exos delivery, PCL is particularly useful as an electrospun fibrous scaffold, wound dressing, or implantable matrix that provides a high-surface-area structure for exosome incorporation and prolongs their local retention at damaged skin sites [116]. However, the hydrophobic and relatively bioinert nature of PCL may limit exosome loading efficiency, homogeneous vesicle distribution, and cell–matrix interactions. Therefore, PCL-based SC-Exos delivery systems often require surface modification, blending with hydrophilic or natural polymers, or incorporation into composite matrices to improve vesicle retention, release behavior, and biological performance [117,118].

#### 3.2.3. Poly(lactic-co-glycolic acid) (PLGA)

PLGA is a biodegradable synthetic copolymer widely used in controlled-release systems because its degradation kinetics can be tuned by adjusting the lactic acid/glycolic acid ratio, molecular weight, end-group chemistry, and formulation geometry. For SC-Exos delivery, the main advantage of PLGA lies in its ability to prolong local vesicle retention and support gradual release through controlled matrix degradation [119,120,121]. This property may help reduce rapid exosome clearance from damaged skin sites and sustain regenerative signaling within the microenvironment of the diseased skin. However, harsh fabrication conditions, organic solvents, and acidic degradation byproducts of PLGA may affect SC-Exos integrity and biological activity [119]. Thus, PLGA-based delivery systems require careful optimization of loading methods and degradation behavior to achieve sustained release while preserving SC-Exos function [122,123,124].

## 4. Engineering Polymer Architectures for Optimized Delivery

**Figure 3:**
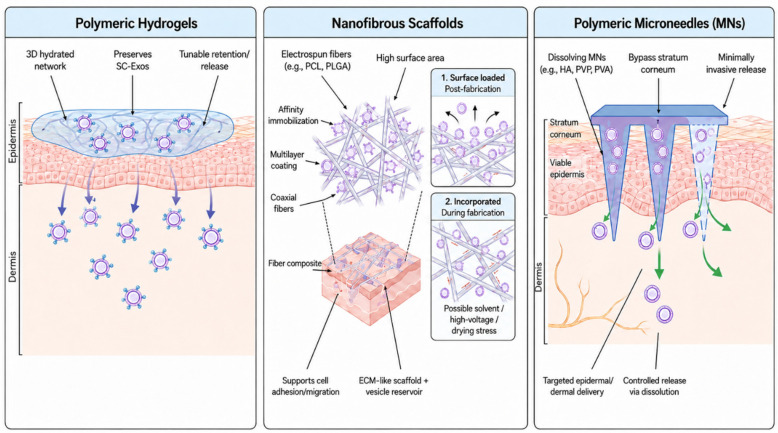
**Engineering polymer architectures for optimized SC-Exos delivery to skin lesions.** Hydrogels, nanofibrous scaffolds, and microneedles represent three major polymer-based formats for SC-Exos delivery. Each architecture offers distinct physicochemical and structural features that can be engineered to address key delivery challenges, including vesicle protection, topical positioning, local retention, and controlled release. By improving SC-Exos localization and bioavailability within diseased skin, these polymeric systems can create tissue-interactive microenvironments that support SC-Exos-mediated skin repair.

The design of polymer-based delivery formats critically determines how SC-Exos are positioned, retained, protected, and released within skin tissue. Hydrogels, nanofibrous scaffolds, and microneedles represent three major polymer-based formats that have been widely explored for SC-Exos delivery [125,126]. By exploiting distinct structural features, these platforms address key delivery challenges, including local retention, wound surface presentation, and transdermal penetration, thereby creating tissue-interactive microenvironments that support SC-Exos-mediated regeneration of damaged skin (Figure 3) [127].

A critical engineering consideration across all polymeric formats is the preservation of exosome post-encapsulation integrity. Specifically, harsh organic solvents and high-voltage electric fields during conventional electrospinning, as well as cytotoxic chemical crosslinkers (e.g., glutaraldehyde) and subsequent drying/lyophilization processes, can severely denature key exosomal transmembrane proteins (e.g., CD9, CD63, CD81) and permanently disrupt their fragile lipid bilayer structures [128,129]. Therefore, employing mild alternatives, such as core-shell coaxial electrospinning or gentle photo-crosslinking, is highly recommended to preserve vesicle functions. Strategies such as ionic crosslinking (e.g., alginate), dynamic covalent chemistry (e.g., Schiff base formation), or core–shell electrospinning (where exosomes are shielded within an aqueous core) are strongly preferred [125]. Furthermore, rigorous post-release validation such as utilizing Nanoparticle Tracking Analysis (NTA), transmission electron microscopy (TEM), and Western blotting is mandatory to confirm that the physical architecture and molecular cargo of the vesicles remain strictly unaltered after encapsulation and subsequent release [38,130].

### 4.1. Polymeric Hydrogels

Hydrogels are three-dimensional crosslinked polymer networks with high water content, allowing them to mimic the proteoglycan- and glycosaminoglycan-rich ground substance of the skin ECM. As hydrated local depots, hydrogels can preserve SC-Exos stability, reduce rapid clearance, and provide a signaling-permissive microenvironment for tissue repair [125,131]. By tuning polymer concentration, crosslinking density, mesh size, swelling behavior, and degradation rate, together with the introduction of exosome-binding motifs, hydrogels can control SC-Exos retention and release through both physical confinement and affinity-based interactions. Injectable hydrogels, which undergo sol–gel transitions in situ via chemical or physical crosslinking, are particularly advantageous because they can conformally fill irregular wound beds and provide intimate contact with inflamed tissue. However, conventional hydrogels also have several limitations for SC-Exos delivery. Their highly hydrated and porous networks may permit rapid vesicle diffusion, particularly when SC-Exos are retained only through weak physical entrapment. This issue can be exacerbated in exudative or enzymatically active wound environments, where excessive swelling or premature degradation may further compromise local retention [132,133,134,135]. Therefore, hydrogel systems for SC-Exos delivery require rational control of network architecture, degradation kinetics, mechanical stability, and vesicle–matrix interactions to achieve optimal SC-Exos retention and release [128,129,136]. Recently, photopolymerization has emerged as a highly active matrix engineering strategy to overcome the potential chemical toxicity of traditional crosslinkers. Utilizing methacrylated polymers, such as gelatin methacryloyl (GelMA) or hyaluronic acid methacrylate (HA-MA), combined with visible-light-activated, cytocompatible photoinitiators, researchers can achieve rapid in situ crosslinking that effectively traps SC-Exos while completely preserving their fragile lipid bilayers and functional transmembrane proteins [137].

### 4.2. Nanofibrous Scaffolds

Nanofibrous scaffolds are polymeric matrices typically fabricated by electrospinning synthetic polymers such as PCL or PLGA. Their interconnected fibrillar structure resembles the collagen- and elastin-rich fibrous component of skin ECM, providing topographical cues for cell adhesion and migration. For SC-Exos delivery, their high surface-area-to-volume ratio and porous fiber network provide abundant interfaces and diffusion pathways for vesicle loading, retention, and release, which can be further controlled by adjusting fiber geometry, surface chemistry, degradation behavior, and loading strategy [138,139]. These features allow nanofibrous scaffolds to perform a dual function: providing ECM-like fibrillar support for tissue repair while enabling localized SC-Exos presentation along fiber surfaces, fiber interiors, or inter fiber spaces [140]. However, nanofibrous scaffold-based SC-Exos delivery still faces loading and fabrication related challenges. When SC-Exos are loaded after scaffold fabrication, this approach may leave many vesicles on outer fiber surfaces or shallow inter-fiber regions, resulting in nonuniform distribution and burst release. Alternatively, incorporating exosomes during fiber fabrication may improve their distribution within the scaffold, but can expose vesicles to organic solvents, high voltage, or drying stress, potentially compromising their integrity and bioactivity. Therefore, affinity-based immobilization, multilayer coating, coaxial fiber design, or hydrogel–nanofiber composite systems can be used to improve vesicle penetration, retention, stability, and sustained release [141,142,143,144].

### 4.3. Polymeric Microneedles (MNs)

MNs are barrier-bypassing polymeric devices that deliver SC-Exos into epidermal or dermal compartments by physically penetrating the stratum corneum, a major barrier to topical delivery that can become abnormally thickened, particularly in lichenified AD skin. This barrier-bypassing capability makes MNs particularly useful for SC-Exos delivery, as nanosized vesicles have limited penetration across the stratum corneum when applied topically. MNs can deposit SC-Exos directly into viable epidermal or superficial dermal compartments, thereby increasing vesicle exposure to therapeutically relevant recipient cells, such as keratinocytes, dermal fibroblasts, endothelial cells, immune cells, and hair follicle-associated niches [145,146,147]. Through controlled needle length and geometry, MNs can access these target compartments while limiting deep stimulation of dermal nerve endings, enabling minimally invasive delivery with reduced pain. Moreover, in dissolving MN systems fabricated from polymers such as HA, poly(*N*-vinyl-2-pyrrolidone) (PVP), or PVA, SC-Exos incorporated within the needle matrix can be released as the matrix dissolves in inflamed skin tissues, enabling direct delivery into an immunologically active microenvironment while allowing release kinetics to be controlled by engineering polymer composition and dissolution behavior [148,149,150,151]. However, MN-based SC-Exos delivery still faces several challenges, including limited vesicle loading capacity, drying-induced vesicle instability, insufficient needle mechanical strength, variable insertion depth, and dose nonuniformity. To address these issues, MN systems require rational optimization of polymer composition, needle geometry, matrix dissolution kinetics, and the incorporation of vesicle-stabilizing agents. In addition, mild fabrication processes, cryoprotectants, and humidity-controlled storage are needed to preserve SC-Exos integrity while ensuring reproducible skin insertion, controlled release, and therapeutic bioactivity [152,153,154]. To comprehensively summarize these engineering strategies, Table 2 compares the diverse polymeric platforms, their loading/release mechanisms, and their relative advantages for SC-Exos delivery.

## 5. Smart Polymeric Systems: Microenvironment-Responsive Release

Smart polymeric systems are designed to sense pathological triggers in ISDs, enabling SC-Exos release kinetics to be tailored to the inflammatory microenvironment and thereby maximizing therapeutic efficacy. Unlike conventional polymeric systems that primarily act as passive depots, smart polymers release their cargos by undergoing stimulus-induced physicochemical changes, such as swelling, dissolution, network relaxation, degradation, or altered vesicle–matrix interactions [125,155,156]. These changes allow SC-Exos release to be more closely coupled to the pathological microenvironment, rather than being governed solely by passive diffusion or matrix degradation. Hydrogels have been the most widely used format for such systems because their hydrated and tunable networks can readily incorporate stimuli-responsive moieties, although similar design principles can also be applied to nanofibrous scaffolds, MNs, and hybrid polymeric platforms [157].

**Figure 4:**
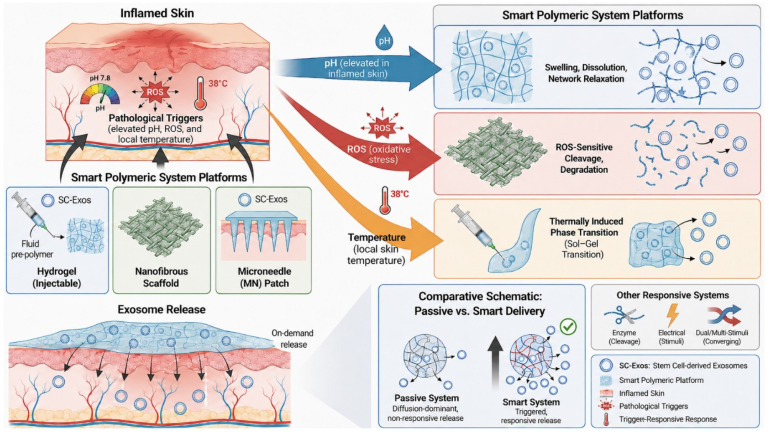
**Smart polymeric systems for microenvironment-responsive SC-Exos release.** Smart polymers are designed to undergo physicochemical changes in response to pathological triggers in ISDs, including elevated pH, increased ROS, and local temperature changes. In the forms of hydrogels, nanofibrous scaffolds, and microneedles, these platforms enable SC-Exos release to be more closely coupled to the lesion-specific microenvironmental cues, rather than being governed by passive diffusion. In this way, smart polymeric platforms allow SC-Exos delivery to be spatially and temporally matched to the inflammatory skin microenvironment.

Among the pathophysiological alterations that can be incorporated into these designs, pH, ROS, and temperature have been most widely investigated as triggers for regulating SC-Exos retention and release. pH-responsive systems exploit the elevated pH observed in inflamed, wounded, or barrier-disrupted skin lesions to alter polymer ionization, causing swelling, dissolution, or network contraction that facilitates vesicle release from the matrix [158,159]. ROS-responsive polymers respond to oxidative stress in inflammatory skin microenvironments through ROS-sensitive cleavage or degradation, thereby promoting SC-Exos release at ROS-enriched lesion sites. Temperature-responsive systems use thermally induced phase transitions to control injectability, in situ gelation, local retention, and release kinetics, and may also take advantage of temperature gradients across skin layers to guide vesicle delivery toward deeper tissue regions. Beyond these major triggers, enzyme-cleavable, electrically responsive, and dual- or multi-stimuli-responsive systems may provide additional control over the timing, location, and extent of SC-Exos release. In this way, smart polymeric platforms can convert lesion-specific biochemical or physical signals into on-demand vesicle release, allowing SC-Exos delivery to be spatially and temporally matched to the pathological microenvironment (Figure 4) [160,161,162].

### 5.1. pH-Responsive Polymeric Systems

pH-responsive polymeric systems are particularly relevant for ISDs because skin pH is closely associated with barrier integrity and disease status. Healthy skin maintains a mildly acidic surface environment, typically around pH 4.5–5.5, whereas ISD-affected or barrier-disrupted skin often shifts toward a less acidic to near-neutral range, approximately pH 5.5–7.0 depending on disease severity, lesion type, and anatomical site [52]. In more severely damaged or chronic wound-like lesions, the local pH can further increase toward neutral to alkaline values, often reported around pH 6.5–8.5 or higher [52]. This pathological pH shift can be exploited as a microenvironmental cue to improve the efficacy of SC-Exos delivery by modulating polymer ionization, matrix swelling, and degradation behavior. In this way, pH-responsive polymers can respond to the altered pH of inflamed or barrier-disrupted skin and translate this disease-associated cue into localized SC-Exos release [159,163].

pH-responsive polymeric systems rely on pH-dependent ionization of polymer functional groups, which is governed by the relationship between local pH and polymer pKa. For ISD-targeted delivery, anionic polymers are particularly suitable when their pKa is matched to the elevated pH range of inflamed or barrier-disrupted lesion (pH 5.5–7.5) rather than that of healthy skin (pH 4.5–5.5) [164]. Under these conditions, weak acidic groups, such as carboxyl groups, become deprotonated and negatively charged, increasing electrostatic repulsion along the polymer backbone and inducing network swelling, matrix relaxation, or partial dissolution [165,166]. These structural changes can enlarge the polymer mesh, facilitate SC-Exos diffusion, and trigger SC-Exos release from the matrix. Representative anionic pH-responsive polymers used in skin-related delivery and tissue engineering include alginate, hyaluronic acid derivatives, pectin, carboxymethyl cellulose, poly(acrylic acid), poly(methacrylic acid), and their modified derivatives [167].

Cationic polymers provide another pH-responsive strategy based on protonation-triggered swelling. These polymers contain amine groups that become positively charged when the local pH falls below their pKa, resulting in charge-driven electrostatic repulsion, and network swelling. This mechanism has been applied to acidic pathological environments, including skin tumors, infection-associated acidic niches, and intracellular compartments such as endosomes and lysosomes, where local acidification can trigger polymer swelling or destabilization and promote cargo release. Thus, cationic systems may be useful for SC-Exos delivery when acidic skin pathology or intracellular acidification is considered as the release cue [168,169].

Overall, pH-responsive polymeric systems can convert local pH abnormalities into controlled SC-Exos retention and release. For inflammatory skin lesions with elevated pH, anionic polymers that undergo deprotonation-induced swelling, relaxation, or dissolution provide the most direct mechanism for vesicle release. Cationic polymers may serve as complementary platforms when their pH-dependent swelling and electrostatic interactions are carefully matched to the target microenvironment. In both cases, the design of pH-responsive matrices should balance ionization behavior, mesh size, degradation kinetics, and vesicle–matrix affinity to promote SC-Exos release while preserving vesicle integrity [170].

### 5.2. ROS-Responsive Polymeric Systems

Reactive oxygen species (ROS), including hydrogen peroxide (H_2_O_2_), superoxide radicals (O_2_•^−^), and hydroxyl radicals (•OH), play a dual role in skin homeostasis. While physiological levels of ROS are essential for cellular signaling and defense against pathogens, the excessive accumulation of ROS is a hallmark of inflammatory skin diseases (ISDs) [171,172]. In chronic lesions of atopic dermatitis and severe wounds, hyperactivated immune cells (such as macrophages and neutrophils) continuously generate high levels of ROS. This prolonged oxidative stress exacerbates local inflammation, induces lipid peroxidation of cell membranes, and degrades extracellular matrix (ECM) proteins, ultimately impeding the healing process [173,174,175]. Furthermore, high ROS levels can compromise the structural integrity of the lipid bilayer of administered stem cell-derived exosomes (SC-Exos), leading to premature degradation and loss of their therapeutic cargo.

ROS-responsive polymeric systems are uniquely designed to leverage this pathological oxidative microenvironment. These polymers incorporate ROS-cleavable linkages such as thioketals, boronic esters, thioethers, and proline-rich peptide sequences into their backbone or crosslinking networks [176]. Upon exposure to elevated ROS concentrations in the inflamed skin, these specific chemical bonds are broken, leading to matrix degradation or significant network swelling. This structural disruption triggers the spatiotemporally controlled release of the encapsulated SC-Exos precisely at the site of severe inflammation [177].

A significant advantage of ROS-responsive polymers is their inherent ROS-scavenging capability. As the ROS-sensitive bonds react with the local oxidants to release the exosomes, they simultaneously deplete the excessive ROS in the microenvironment [178]. This dual-action mechanism not only facilitates ‘on-demand’ exosome delivery but also simultaneously depletes the excessive ROS in the microenvironment, thereby directly alleviating oxidative stress and synergistically resolving chronic tissue inflammation while preserving vesicle bioactivity [125].

### 5.3. Temperature-Responsive Polymeric Systems

Temperature-responsive (thermosensitive) polymers offer a highly practical approach for SC-Exos delivery, capitalizing on the distinct temperature differences between ambient storage conditions and the physiological environment of the skin (approximately 32–35 °C for normal skin, potentially higher in inflamed lesions). These systems undergo a rapid, reversible sol-gel phase transition around their lower critical solution temperature (LCST) [179,180,181].

Below the LCST (e.g., at room temperature), the polymer remains in a flowable liquid state, allowing for the homogeneous mixing of SC-Exos without exposing them to harsh crosslinking chemicals or physical stress. This liquid state also enables the formulation to be easily injected or conformally applied to irregularly shaped wound beds and extensive ISD lesions [112,180,181]. Upon application to the skin, the physiological temperature triggers immediate in situ gelation, forming a stable hydrogel network [89].

Representative temperature-responsive polymers include Pluronic F127 (Poloxamer 407), PNIPAM, and specific chitosan–glycerophosphate blends [182,183]. By utilizing these systems, SC-Exos are robustly immobilized at the target site, significantly minimizing burst release and preventing the formulation from being washed away by wound exudates. The resulting gel acts as a sustained-release depot, allowing exosomes to diffuse steadily into the dermal layers as the polymer gradually degrades, thus ensuring a prolonged therapeutic window [183,184].

## 6. Synergistic Therapeutic Mechanisms in Skin Regeneration

The integration of SC-Exos within polymeric biomaterials is not merely a vehicle-cargo relationship; it represents a highly synergistic therapeutic paradigm. When successfully delivered into the ISD microenvironment, the complex interplay between the engineered polymer matrix and the exosomal cargo collaboratively orchestrates tissue repair at multiple biological levels. It is critical to distinguish between mere additive effects and true synergism in polymer–exosome systems. An additive effect occurs when the polymer and the exosomes operate independently without enhancing each other’s intrinsic bioactivity. In contrast, true synergism arises from an interdependent relationship where the polymer matrix actively remodels the physical niche (e.g., via hydration or providing a provisional ECM), significantly increasing the receptivity of target cells to the SC-Exos’ microRNA cargo, which collectively amplifies downstream regenerative and immunomodulatory pathways, such as M2 macrophage polarization.

### 6.1. Immunomodulation and Macrophage Polarization

SC-Exos are rich in anti-inflammatory microRNAs (e.g., miR-146a, miR-21) and proteins that actively suppress pro-inflammatory cytokine cascades (TNF-α, IL-1β) [184,185,186]. A critical mechanism is their ability to drive the polarization of local macrophages from the pro-inflammatory M1 phenotype to the pro-regenerative M2 phenotype. When delivered via stimuli-responsive hydrogels, the sustained release ensures continuous immunomodulation, preventing the relapse of the inflammatory phase often seen in chronic ISDs [113,183,187].

### 6.2. Angiogenesis and Re-Epithelialization

SC-Exos promote the proliferation and migration of local keratinocytes and human umbilical vein endothelial cells (HUVECs), driven by exosomal growth factors (such as VEGF and basic FGF). Concurrently, structural polymers (like collagen or nanofibrous scaffolds) provide the essential physical cues and 3D architecture for these cells to adhere and migrate, accelerating the formation of a robust vascular network and complete epidermal closure [188,189,190,191].

### 6.3. Barrier Restoration and ECM Remodeling

The intrinsic properties of natural polymers contribute directly to barrier repair. HA provides deep tissue hydration, crucial for alleviating the severe dryness and pruritus in AD skin. Chitosan offers a broad-spectrum antibacterial shield, preventing secondary infections [90,192]. As the polymer provides a provisional matrix, the SC-Exos stimulate dermal fibroblasts to synthesize native collagen and elastin, promoting proper ECM remodeling and minimizing scar tissue formation [193].

## 7. Current Challenges and Future Perspectives

### 7.1. Exosome Batch Standardization and Quality Control (QC)

A primary translational barrier is ensuring batch-to-batch consistency. Following the MISEV guidelines, rigorous QC parameters—including isolation methodology, precise particle size distribution, purity tracking, and quantification of specific surface markers (CD9, CD63)—must be standardized before integrating SC-Exos into polymeric matrices.

### 7.2. Large-Scale Manufacturing and Storage Stability

Do the electrospinning, crosslinking, and drying processes deteriorate exosome functions? The integration of exosomes into polymers during large-scale manufacturing often exposes them to drying stress and solvent toxicity. Implementing advanced cryoprotectants and lyophilization techniques is essential to maintain long-term storage stability without collapsing the vesicle lipid bilayer.

### 7.3. Future Perspectives

Future research should focus on refining the fabrication processes, such as utilizing microfluidics for scalable and highly uniform polymer–exosome nanoparticle generation, and developing combinatorial platforms (e.g., dissolving microneedles integrated with smart hydrogels) that can non-invasively penetrate the stratum corneum and deposit stimuli-responsive depots directly into the dermis.

Furthermore, a significant biological limitation lies in the current preclinical evaluation models. Most in vivo studies evaluating polymer–exosome platforms rely heavily on rodent models. However, murine skin fundamentally differs from human skin in terms of epidermal thickness, hair follicle density, immunological profiling, and the primary mechanism of wound closure (which is primarily driven by contraction in rodents versus re-epithelialization in humans). To accurately predict clinical efficacy, immune compatibility, and degradation kinetics of smart polymeric systems, future studies must transition towards more clinically relevant models. The utilization of porcine models, which closely mimic human skin biomechanics, or advanced 3D human skin equivalents and organoids that can faithfully replicate the human neuro-immune-cutaneous axis, will be crucial steps in translating these polymeric nanomedicines from the bench to the bedside.

## 8. Conclusions

The convergence of advanced polymer science and cell-free exosome therapy presents a transformative approach for the treatment of inflammatory skin diseases and severe wounds. While stem cell-derived exosomes offer unparalleled regenerative and immunomodulatory properties, their clinical utility is inherently limited by environmental instability and rapid clearance. As highlighted in this review, the strategic engineering of polymeric biomaterials—ranging from natural and synthetic hydrogels to nanofibrous scaffolds and microneedles—effectively overcomes these physiological barriers. Furthermore, the advent of smart, microenvironment-responsive polymers enables disease-triggered, on-demand release of therapeutic vesicles. By not only protecting SC-Exos but also acting synergistically to repair the skin barrier and guide cellular behavior, these sophisticated polymeric platforms hold immense promise for revolutionizing the clinical management of chronic and inflammatory dermatological conditions.

## Figures and Tables

**Table 1:** Comparative overview of therapeutic components and application methods for skin diseases.

Therapeutic Strategy	Representative Therapeutic Components/Materials	Therapeutic Effects	Advantages	Disadvantages	References
**Topical** **anti-inflammatory** **therapy**	Topical corticosteroids, calcineurin inhibitors, PDE4 inhibitors, topical JAK inhibitors, vitamin D analogs, retinoids	Suppresses local inflammatory signaling and reduces disease flare at skin lesions.	Established efficacy; lesion-directed treatment	Long-term safety concerns for several agents; recurrence after discontinuation; limited regenerative activity	[16,17,18]
**Systemic** **immunomodulators**	Biologics targeting IL-4/IL-13, IL-17, IL-23, TNF-α, IgE, or related pathways; oral JAK inhibitors; selected PDE4 inhibitors	Controls moderate-to-severe widespread inflammation through pathway-specific immune modulation.	Pathway-specific targeting; clinically validated in major ISDs	High cost; systemic adverse effects; limited regenerative activity	[19,20,21,22,23]
**Phototherapy**	Narrowband UVB, UVA1, PUVA, excimer laser	Modulates local immune responses; can support repigmentation or remodeling.	Localized immunomodulation without biologic modality; combinable with topical approaches; clinically established in selected ISDs	Dose-dependent irritation or burn risk; limited molecular specificity	[19,24,25,26,27]
**Platelet** **concentrate-based** **therapy**	Platelet-related plasma (PRP) and platelet concentrates	Provides endogenous growth factor/cytokine mixtures that can support tissue repair, angiogenesis, and immunomodulation.	Multiple endogenous regenerative mediators	Donor-to-donor variability; limited control over preparation protocols, exact molecular composition, and potency	[28,29]
**Cell transplantation**	MSCs, ADSCs, skin-derived progenitor cells, fibroblasts, keratinocytes	Provides living cells capable of secreting trophic factors, modulating inflammation, supporting ECM remodeling, and promoting tissue repair.	Immunomodulatory and regenerative potential; possible sustained secretion of trophic factors from transplanted cells	Cell survival and engraftment issues; tumorigenicity/immunogenicity concerns; regulatory burden	[30,31,32]
**Conditioned medium (CM) therapy**	MSC-CM, ADSC-CM, UCMSC-CM	Delivers cell-derived paracrine factors without transplantation	Alternative to direct cell transplantation; multiple regenerative and anti-inflammatory mediators	Batch variability; limited standardization and potency assays	[33,34]
**Exosome-based therapy**	Stem cell-derived EVs/exosomes	Provides cell-free multi-cargo signaling for immunomodulation, barrier repair, angiogenesis, fibroblast/keratinocyte regulation, and ECM remodeling.	Cell-free; lower risk than direct cell transplantation; multi-target paracrine activity	Limited clinical standardization; isolation/storage challenges; stability and scale-up issues;	[35,36,37,38]

**Table 2:** Comparative overview of Polymeric delivery platforms for SC-Exos targeting skin repair and regeneration.

Delivery Format	Polymer Types	Exosome Loading Method	Release Mechanism	Exosome Protection Mechanisms	Mechanical Property	Key Advantages	Major Limitations & Challenges	Applications	References
Natural Hydrogels	Hyaluronic Acid (HA)	Post-loading of exosomes onto preformed hydrogel scaffolds [80,81]; Physical mixing before gelation [82,83]	Matrix swelling, diffusion, and degradation	Physical entrapment; reduced dehydration and rapid washout; protection from local proteolytic/oxidative stress	Soft and tissue-compatible; mechanically reinforced with additional crosslinking	ECM-mimetic highly hydrated network; high biocompatibility; HA receptor-mediated cell interactions	Relatively low mechanical strength; Rapid in vivo degradation	Diabetic ulcer [80]; Chronic wound [82,83]	[80,81,82,83]
	Chitosan	Post-loading of exosomes onto preformed hydrogel scaffolds [85]; Physical mixing before gelation [83,84,88,89,90,91,92]	Matrix swelling, diffusion, ion-exchange, and degradation	Enhanced retention via electrostatic interaction; indirect antimicrobial/anti-inflammatory support	Bioadhesive and flexible; mechanically reinforced with polymer blending or additional crosslinking	Rapid in situ gelation; hemostatic properties; intrinsic antibacterial activity; electrostatic cell adhesions	Poor solubility and mechanical strength in acidic conditions	Diabetic wound [85,88,107]; Diabetic ulcer [89]; Acute/chronic wound [83,84,90,91,92];	[83,84,85,88,89,90,91,92,107]
	Alginate	Post-loading of exosomes onto preformed scaffolds [102]; Physical mixing before ionic crosslinking [90,94,97,103,116]	Matrix swelling, diffusion, ionic crosslink dissociation, and degradation	Physical entrapment and protection; reduced washout	Soft and highly hydrated; mechanically strengthened by additional ionic crosslinking or blending with other polymer matrices	Ionic gelation in mild conditions; highly hydrated; biocompatibility; rapid in situ gelation	Limited mechanical strength; Lack of cell adhesion; Lack of mammalian enzymatic degradation; Ion exchange-dependent instability and burst release risk	Acute/chronic wound [90,94,102,103,116]	[90,94,97,102,103,116]
	Collagen	Physical mixing before ionic crosslinking [91,104,107]	Matrix swelling; diffusion, network relaxation, and degradation	Physical entrapment and protection; reduced washout	Fibrillar and ECM-like matrix; mechanically strengthened by crosslinking or composite reinforcement	Native ECM component; high biocompatibility; promoting Integrin-mediated cell adhesion, migration, and ECM remodeling	Rapid enzymatic degradation; weak mechanical stability; potential batch variability or immunogenicity depending on collagen source	Acute/chronic wound [91,104]; Diabetic wound [107]	[91,104,107]
	Gelatin (GelMA)	Mixing exosomes with precursor solution and crosslinking [98,99,100,101]	Matrix swelling, diffusion, network relaxation, and degradation	Physical entrapment and protection; reduced washout	ECM-like properties; relatively tunable stiffness	Cell-adhesion motifs; supporting cell migration and ECM remodeling	Rapid enzymatic degradation; crosslinker-mediated vesicle or cellular stress; batch variability from gelatin source	Acute/chronic wound [100,101]; Diabetic wound [98]; Hypertrophic scar [99]	[98,99,100,101]
Synthetic Hydrogels	PEG [92,112,113,115], PVA [97,132,133], PNIPAM [134], PAA [135]	Pre-gel mixing before gelation [97,113,115,133,135]; Post-loading into preformed hydrogel scaffolds [134]; Nanocarrier-assisted incorporation before gelation [132]; Pre-gel mixing in thermosensitive hydrogel precursor [92,112]	Diffusion-controlled release; Stimuli-responsive degradation or network transition in response to pH, ROS, or temperature	Tunable crosslinked polymeric networks supporting physical protection and vesicle retention	Highly tunable stiffness, elasticity, swelling, and degradation behavior.	Highly tunable mechanical and rheological properties; batch-to-batch consistency; stimuli-responsive release capabilities.	Lack of intrinsic cell-adhesive motifs; potential cytotoxicity from unreacted chemical crosslinkers	Acute/chronic wound [92,112,113,132,135]; Diabetic wound [97,115,133,134,135]	[97,112,113,115,132,133,134,135]
Nanofibrous Scaffolds	PCL [116,118,143], PLGA [118,124], PLLA [144]	Post-loading onto electrospun scaffolds [116,118,144]; Coaxial core–shell electrospinning [124,143]	Surface desorption; Gradual hydrolytic degradation of polymer backbone	Reduced rapid washout	ECM-like fibrous support with relatively high mechanical stability.	High surface-area-to-volume ratio; structural support replicating fibrous skin ECM; sustained release over weeks.	Possible damage from harsh organic solvents and high voltage during electrospinning	Acute/chronic wound [116,143]; Diabetic ulcer [118]; Diabetic wound [144]	[116,118,124,143,144]
Polymeric Microneedles (MNs)	Dissolving polymers, including HA, PVP, PVA [150,151]	Casting and micromolding of polymer–exosome mixture [150,151]	Matrix dissolution upon contact with interstitial skin fluid	Polymer matrices can stabilize exosomes during localized delivery	Strong enough for skin insertion and designed to dissolve or swell after penetration.	Bypasses the stratum corneum physically; painless and minimally invasive targeted dermal delivery.	Limited exosome loading capacity per patch; drying stress during fabrication can compromise vesicle stability.	Diabetic wound [150,151]	[150,151]

## Data Availability

No new data were created or analyzed in this study. Data sharing is not applicable to this article.

## Abbreviations

AD	Atopic dermatitis
ECM	Extracellular matrix
GelMA	Gelatin methacryloyl
HA	Hyaluronic acid
ISD	Inflammatory skin disease
MMP	Matrix metalloproteinase
MN	Microneedle
PCL	Poly(ε-caprolactone)
PEG	Poly(ethylene glycol)
PLGA	Poly(lactic-co-glycolic acid)
PNIPAM	Poly(*N*-isopropylacrylamide)
PVA	Poly(vinyl alcohol)
ROS	Reactive oxygen species
SC-Exos	Stem cell-derived exosomes
